# Cutting-edge biotechnological advancement in islet delivery using pancreatic and cellular approaches

**DOI:** 10.2144/fsoa-2020-0105

**Published:** 2020-11-23

**Authors:** Magdy Elnashar, Mauro Vaccarezza, Hani Al-Salami

**Affiliations:** 1 Biotechnology & Drug Development Research Laboratory, School of Pharmacy & Biomedical Sciences, Curtin Health Innovation Research Institute, Curtin University, Perth, Western Australia, Australia; 2 Centre of Excellence, Department of Polymers, National Research Centre, Cairo, Egypt; 3 School of Pharmacy & Biomedical Science, Faculty of Health Sciences, Curtin University, Bentley, Perth, WA 6102, Australia

**Keywords:** auto-transplantation, bile acid, diabetes treatment, encapsulation, human pancreas, pancreatic islets, stem cells, transplantation, Type 1 diabetes

## Abstract

There are approximately 1 billion prediabetic people worldwide, and the global cost for diabetes mellitus (DM) is estimated to be $825 billion. In regard to Type 1 DM, transplanting a whole pancreas or its islets has gained the attention of researchers in the last few decades. Recent studies showed that islet transplantation (ILT) containing insulin-producing β cells is the most notable advancement cure for Type 1 DM. However, this procedure has been hindered by shortage and lack of sufficient islet donors and the need for long-term immunosuppression of any potential graft rejection. The strategy of encapsulation may avoid the rejection of stem-cell-derived allogeneic islets or xenogeneic islets. This review article describes various biotechnology features in encapsulation-of-islet-cell therapy for humans, including the use of bile acids.

## Lay abstract

The global cost for diabetes mellitus (DM) is estimated to be $825 billion. With regard to Type 1 DM, transplanting pancreas whole/islets have gained the attention of researchers in the last few decades. Recent studies have demonstrated that islet transplants containing insulin-producing β cells is the most notable advancement cure for Type 1 DM. However, this procedure has been hampered by islet donor shortage and the need for long-term immunosuppressive therapy. An encapsulation strategy could overcome these shortcomings. Here, we discuss various biotechnology features in the encapsulation of islet cell therapy for humans including the use of bile acids.

According to the World Health Organization, diabetes occurs when the pancreas produces too little insulin for the body's requirements, or when the body cannot use insulin effectively. Chronic diabetic complications impair the metabolic processing of fats, carbohydrates and electrolytes, which disrupts the vascular system [1]. Examples of microvascular complications are myocardial infarction and retinopathy (e.g., blindness), and for vascular complications are nephropathy and neuropathy (e.g., foot amputation). Type 2 diabetes mellitus (T2DM) is the most prevalent (∼85%), and it occurs due to tissue resistance or lack of insulin. These issues can be managed by lifestyle changes (physical exercise + diet), insulin, oral antidiabetic drugs and noninsulin injections. Patients suffering from T1DM are insulin-dependent, and rely on insulin injection.

The number of diabetic patients dramatically increased from 1980 to 2014, from 108 to 422 million [2] and between 2012 and 2015, there were 3.8 million diabetes-related deaths. That number could increase greatly, as there are now approximately one billion pre-diabetic people. The diagnosis and treatment of T1DM is more costly than T2DM, and the global cost for DM is estimated to be $825 billion [3,4].

Despite ongoing research in multiple laboratories worldwide, islet transplantation (ILT) remains confined to clinical trials rather than common practice, using robust commercially available therapeutics. The latest studies on transplanting the pancreas (whole organ or islets) in patients with T1DM with severe glycemic variability found it possible to restore the endocrine functions of the pancreas [5]. Combining total pancreatectomy with islet autotransplantation was another strategy [6]. The application of bile acids (BAs) in islet encapsulation and transplantation has gained interest, and laboratory results show significant promise, not only in maintaining high cellular viability but also rejuvenation postencapsulation, cell bioenergetics, insulin release and cell-cell interaction necessary for acceptable performance, short and long term [7,8].

Recent advancements in the treatment/management of T1DB also include encapsulated pancreatic islets and encapsulated mesenchymal stem (MSCs), as shown in Figure 1.

**Figure 1.**
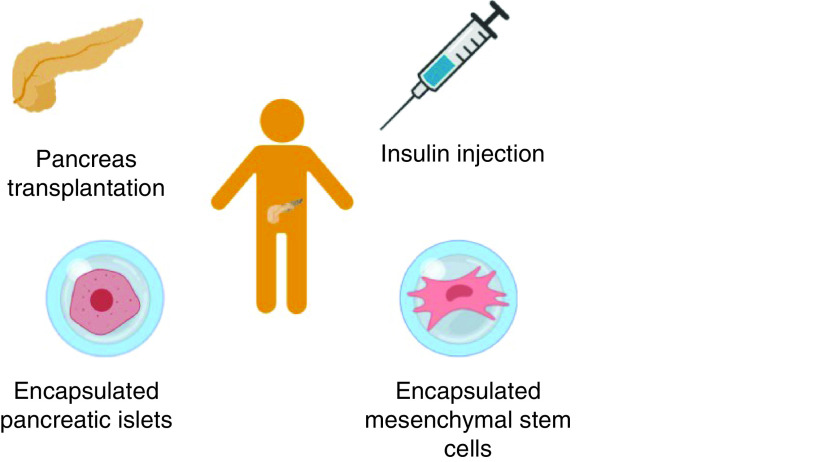
Possible strategies for the treatment and management of Type 1 diabetes mellitus: insulin injection, pancreas transplantation, encapsulated pancreatic islets and encapsulated mesenchymal stem cells.

## Pancreatic cell transplantation compared with whole-pancreas transplantation

### Pancreas transplantation

Transplanting the whole pancreas from a healthy donor to a diabetic patient requires major surgery. The process is invasive, and over the past 30 years, research activity has led to some standardized protocols to achieve this process without increasing the rate of hypoglycemic events in the recipient [9–12]. Since 1966, the rate of implant postsurgery survival has been well maintained at 76% after 1 year, 62% after 3 years and 50–70% after 5 years [13]. Over the last decade, the number of solitary operations has remained stable with a steady rate of graft failure, especially in the last 5 years [14].

### Limitations of pancreatic transplantation

According to Ramesh & Brayman, the procedure of pancreatic transplantation (PCT), which is generally performed concurrently with kidney transplantation, includes some surgical and postsurgical complications. The major complications involve graft pancreatitis/thrombosis, and formation of pseudocysts and pancreatic fistulae [15]. Finding a donor is another issue.

### Islet transplantation

 ILT comes as an alternative, and is considered as an upgraded way to treat T1DM, in contrast to injection with insulin or whole-pancreas transplantation. It requires approximately 2% (w/w) of the total pancreas (endocrine part) that contains β cells in a healthy person to be transplanted into a diabetic person with reasonable success. ILT can be infused using a catheter through the portal venous access, which represents a minimally invasive process and is also associated with marginal or no complications [16]. Another positive aspect of ILT is that it can deliver glycemic control without the risk of hypoglycemia and the use of exogenous insulin. For instance, Shapiro has explored ILT and its potential use, and has demonstrated that ILT does possess the ability and validity to correct HbA1c concentrations to certain values that may reverse the secondary consequences of diabetes [17], while insulin administration via pump or similar set-ups may result in fewer or attenuated effects on concentrations of the diabetic biomarker HbA1C [18]. Thompson *et al.* found that ILTs are more effective in decreasing the progression of microvascular diseases (e.g., retinopathy) and vascular diseases (e.g., nephropathy) related to diabetes than is rigorous medical therapy [19]. Although this method is effective, seems simpler and less invasive, it does have some limitations.

A recent study of the Clinical Islet Transplantation (CIT) Consortium protocol 07 (CIT-07) has shown the results in 48 adults with T1D for more than 5 years [20–22]. Patients were suffering from severe hypoglycemic events (SHEs) and impaired awareness of hypoglycemia (IAH). Purified human pancreatic islets (PHPI) were transplanted at eight centres in North America, and each patient had been administered an immunosuppressive and PHPI. The median HbA1c level was 5.6% at 1–2 years with no death or disability noted [21]. The overall effectiveness of the CIT-07 trial [20,22] has been further demonstrated by the results by Foster *et al.*, showing that we can potentially maintain blood glucose control without experiencing severe cases of hypoglycemia, and hence improved quality of life via successful ILT that is safe and robust [21]. Of note, large decreases in diabetes-related distress and fear of hypoglycemia were observed. Furthermore, quality of life and functional health status did not worsen, despite ongoing immunosuppression. General measurements of health status, such as Short Form 36 Health Survey (SF-36) and mental summary score (MCS), demonstrated statistically significant differences from baseline status. The CIT-07 result gives a new perspective on ILT: a prospective assessment of health utility and health status outcomes among transplant recipients in a Phase-III study.

### Limitations of ILT

Long-term causes of graft loss can be listed as:

#### Immunosuppression-associated factors

Patients with ILT are normally prescribed immune-suppressive medications (systematic immunosuppressive treatment) to avoid allogeneic rejections. Immunosuppressive medication should be taken in optimized doses to avert graft loss. However, even the optimized medications can be directly toxic to the ILT or cause dysfunction of other tissue organs after long-term usage [23]. In addition, systemic immune-suppressive medications such as cyclosporine and tacrolimus may also increase the patient's risk of developing cancer, organ damage or infection. Histological studies found that ILT triggers recurrent autoimmune effects that can result in cell destruction [24,25]. The use of a specific antigen to inhibit the immune response through the initiation of regulatory T cells, has shown some promising results in avoiding the development of the response [26]. However, in the USA from 1999 to 2007, less than 400 ILTs were performed out of ∼1.25 M cases nationwide, and that was linked to the high risk of chronic immunosuppression which presently outweighs the potential benefits of ILT [27,28].

#### Nonimmunosuppression-associated factors

ILTs are engrafted in the liver through the portal system, and the process may encounter islet-graft loss due to hypoxia and/or instant blood-mediated inflammatory factors (INMIR), which may lead to reduced islet mass and poor islet quality and function.

- Hypoxia [29]
  Islet cells are damaged due to hypoxia, as the process of isolation causes de-vascularization in the islet cells. Also, islets are implanted into the liver in low oxygen tension (Carlsson *et al.*). There is the indirect effect of the hypoxic environment stimulating the innate immune system, resulting in the release of the inflammatory cytokines interferon (IFN), IL-1 and TNF [30].
- Instant blood-mediated inflammatory reaction (IBMIR) [31]
  IBMIR causes activation of the coagulation system, resulting in serious problems by disruption [32] and destruction of 60% of transplanted islets [33]. In general, poor clinical outcomes of ILT are frequently related to high IBMIR [30].

The expertise in both ILT and pancreas transplantation is not widely available. This issue renders problematic any decision in regard to therapy, based on the pros and cons of islet versus pancreas transplantation. As a result, diagnosis and management of patients with clinical diabetic symptoms has been guided mostly by local expertise [5]. Recently, Maffi and Secchi, have proposed a protocol based on Shapiro *et al.* [34] to define when to carry out islet or pancreas transplantation and in many scenarios, there was a degree of overlap. Pros and cons for pancreas versus ILT are summarized in Table 1.

**Table 1.** Comparison between the pros and cons of whole-pancreas transplantation versus islet transplantation.

	Islet transplantation	Pancreas transplantation
Biological cost (organ consumption)	Needs several donors	Needs one donor
Biological cost (technical complications during follow-up)	No major complications (∼3.1%)	Major complications (∼10%): bleeding, thrombosis, morbidity and duodenal leaks
Insulin-independence	Delayed process (months-years). 44% is achieved at 3 years	Speedy process. 61% is achieved at 3 years
Surgical complications	No risk	High risk (e.g., pre-existing of cardiovascular disease)

In summary, they suggest that ILT is preferred for patients with:

- Severe glycemic problems that cannot be controlled using insulin therapy
- Unstable Type 1 diabetes
- Hypoglycemia unawareness
- Cardiovascular disease

The most notable advancement cure for T1DM is ILT containing insulin-producing β cells [35,36]. ILT has shown the potential to be one of the most promising solutions for the treatment of T1DM, and that has encouraged many ILT Centres to start license applications for clinical allogeneic ILT. The inflammation of pancreatic β cells and the paucity of cell viability after ILT are the biggest challenges to researchers [37–39].

### Total pancreatectomy & islet autotransplantation

Another potential possible therapeutic approach could be islet autotransplantation which considers only patients with chronic pancreatitis (CP), and is usually preceded by total removal of pancreas (pancreatectomy) [6]. Recent observations by Berman *et al.* show that diabetic candidates with severe painful CP are valid candidates for pancreatectomy [40]. This observation is of paramount significance, especially among candidates and patients with autogenous pancreatic islet transplantation (TP-IAT), in that it has not been readily accessible until recent years before which patients were reported to transplant centres during later stages of their disease. While there has been a growing utilization of TP-IAT for patients with refractory CP over many years, there remains a lack of consensus clinical guidelines to inform the counselling and management of patients undergoing TP-IAT and its eventual utilization as a valuable method to control diabetes and glycemia.

As highlighted recently by Al-Sofiani *et al.*, there is a need to develop uniform practice guidelines and to standardize clinical measurement protocols on how to assess patients before and after surgery; moreover, future research should improve islet isolation and engraftment techniques, and validate new biomarkers and imaging tools able to monitor the function, viability and location of the islet cells after engraftment [6,41]. The future could be even more promising in a similar scenario with encapsulated ILT, avoiding immunosuppressive drug treatment (non-systematic immune-suppressive treatment).

### Nonsystematic immune-suppressive treatment

Nonsystematic immunosuppressive (NSI) treatment uses different approaches to reduce local inflammation and generate immune-privileged sites, for example by engineering materials that release factors (prostaglandins [PGE2], IL-10, transforming growth factor-b, SDF and chemokine MCP1) to reduce inflammation [42–44]. This approach also includes stem cell (autologous β cells), immunomodulation (antigen-specific T cells), immune-protective devices, coatings and capsules [45–47]. Immunoprotection using engineered materials such as encapsulation of ILT has received the greatest consideration.

### Bio-nano- & micro-encapsulation of pancreatic islets

Encapsulation of cells is one way to protect these viable cells from the host immune system, post-transplantation. Islet bio-nano- and micro-encapsulation can be carried out by encapsulating the viable, living and functional components within various forms of semipermeable cases/membranes [48–51]. Encapsulation is the ability of the capsule to envelop the cells/islets and protect them from being recognized/identified by the body's own immune system. Entrapment of pancreatic ILT has been widely researched and aimed to protect the pancreatic islets from the environment and to reduce β cells inflammation and death [52,53]. Encapsulation embraces the approach of isolation and shielding of the islet-grafts from the recipient's immune system, which is different to the conventional strategy of ongoing immunosuppressive treatments. That potentially represents a suitable approach for effectively supporting graft functionality and survival [54].

However, it is obvious that implanting and/or encapsulating biomaterial may still trigger a foreign-body reaction, and may affect the safety of the implanted device. This caveat can considerably impact the short-/long-term tissue responses comprising proteins, cells and other biological components used in regenerative medicine or tissue engineering. Anderson *et al.* [55] have studied the mechanism of foreign-body reaction and the effect of adherent macrophages on the overall inflammatory response to biomaterials. They found that surface chemistry can influence the behavior of macrophages such as cytokine secretion, adhesion, fusion and apoptosis.

In order to encapsulate viable cells or biologically active components including organoids, specialized biomaterials are needed. These biomaterials need to provide support as well as biocompatibility to the encapsulated active moieties including pancreatic islets. The biomaterials need to exhibit a wide range of features including:

■ Being porous enough to faciliate and allow nutrients and oxygen permeation into the graft, and also allow waste to leave the graft
■ Having the capacity to encapsulate high concentrations of the target molecule
■ Acting as an immunostatic or biological barrier to control its core active moieties, while protecting the functionality and performance of the graft
■ Being available and from a reliable source, ready for scale-up production
■ Having good physical and mechanical stability and regularity
■ Not allowing fibrotic growth and subsequent graft failure
■ Maintaining long-term cell viability

Scharp and Marchetti have published a detailed review article on the encapsulation of ILT. The review article comprises different types of capsules/materials and devices [47].

### Preparation of macro-/micro-capsules

The most common size and shape for islet entrapment and encapsulation are in the form of disks or tablets, beads or, less commonly, fibers. Gel microcapsules are the most commonly used form of islet encapsulation, since nano-capsules would ultimately provide a huge surface area and more islet interaction with the outside microenvironment. The tiny micro- and nanocapsules can be fabricated by a wide range of technologies such as the Innotech Encapsulator, ionic-gelation methods, Vibrational Jet-Flow Technology, dripping and interphase technique method [56–58]. The Buchi-supported systems have the advantage of strong and comprehensive control over various encapsulating parameters including production rate, being 50–3000 beads/second, based on the blend of rheological and non-Newtonian parameters [59].

## Biopolymers commonly researched for islet encapsulation

### Hydrogels & water-soluble polymers

There is a wide range of various biomaterials and biopolymers currently available for encapsulation of biomolecules, including hydrogels such as alginate, chitosan, cellulose, hyaluronic acid (HA), collagen and carrageenan [60–75].

### Alginate-based capsules

Among all these hydrogels, alginate has been dominating, and is the most commonly used hydrogel for encapsulation of ILT due to its low cost, high availability and durability, nontoxicity to host organisms and its mechanism for encapsulation is well-established [76–78]. Multiple polymers such as alginates and the like have the appropriate properties to form suitable matrices when multivalent cations such as Ca^2+^ in aqueous medium are present [57,79].

Encapsulation of cells using alginate has been proven to be efficient [80]. For example, Ludwig *et al.* and Prochorov *et al.* have introduced heterologous islets without the use of immunosuppressive drug protocols [81,82]. The main message from these data is the importance of cell viability in the islet graft, which greatly influences the outcomes of the procedure. In particular, the proposed device by Ludwig *et al.* couples an alginate immune-isolated preparation with an oxygenated chamber system. Oxygenation is pivotal for cell survival and correct cell metabolism: in fact, the graft was effective and well-tolerated without immunosuppression. This contribution demonstrates that encapsulation with regulated/controlled oxygenation (avoiding hypoxia at the level of the encapsulated cells) could be a viable option to improve the efficacy of encapsulated ILT without the use of immunosuppressive drugs. It is worth noting that the use of this oxygenated alginate-encapsulation strategy was applied to allogeneic human ILT in humans for the first time by Ludwig *et al.* [82].

In another example by Jacobson-Tulleneers-Thevissen *et al.*, they implanted alginate-encapsulated human islet cells into the peritoneal cavity of mice. The results showed that intraperitoneal transplantation had a better result compared with the free implants under the kidney capsule [83]. Free-floating capsules in the peritoneum, without direct contact with host tissue or metabolic correction, and immediately corrected hyperglycemia, were maintained until the end of the study. Recovered floating capsules demonstrated excellent cell viability and secretion, without signs of inflammation/fibrosis in the recipient rodents. These results prompted the authors to translate the concept in a pilot study run on one T1D patient: the main goal of this study was to assess the survival and function of β cells in the encapsulated environment transplanted in the peritoneum. The function and efficacy were confirmed (as in the rodent study), but signs of fibrosis were present, and most of the capsules were found together in large clumps, sticking to the abdominal wall [83].

However, alginate micro- or nanocapsules alone, without any additives, do not seem to act as good-quality micro- and nano-capsules, given their weak physical stability and loss of the mechanical resistance to shear force over long periods of time, that is required and expected post-transplantation [59,84,85]. This is possibly due to alginate's poor ability to adhere to the crosslinking processes and not having sufficient interactive force to form a solid surface resistant to damage or stress [86]. The literature also suggests that alginate gels are not stable in various types of buffers [87], and like other seaweeds, they are susceptible to fibrotic overgrowth when used *in vivo*, resulting in necrosis of the encapsulated cells and pre-mature graft failure [78,88–90]. Many modifications have been made to improve alginate capsules such as ultra-purification, co-encapsulation and surface treatment, but the final results are not yet satisfactory enough to be used at the clinical level [77,89,91–93].

### HA-based capsules

HA, on the other hand, and especially HA-based hydrogels, have shown to be very suitable for cell-therapy applications and tissue engineering because of their distinctive biological and mechanical properties [94–96]. Formulations based on HA and other polymers, such as denatured collagen (DCOL), have been found to be durable, with a high shear strength of up to 3500 Pa. A commercial version of HA-DCOL is known as HyStem-C, and it has been used *in vivo* to repair osteochondral defects in rabbits and myocardial infarcts in SCID mice [97–99]. HA is used as a replacement for alginate for the encapsulation of ILT, especially to address graft failure associated with fibrosis [88].

### Biomimetics of transplantable materials

Biomimetic materials are hybrids/composites of natural materials (e.g., peptides, amino acids, saccharides and proteins) developed using motivation from nature. They have a lower probability of inducing fibrotic growth and higher biocompatibility, which make them suitable for cell encapsulation [100,101]. Unfortunately, these advanced biomimetic materials have low durability and a high degradation rate, which make them unsuitable for ILT.

### BAs as anti-inflammatory compounds

BAs formed from cholesterol catabolism and are metabolically active compounds. The process of bile-acid synthesis involves two main biological pathways and the metabolic and enzymatic activation of approximately 17 hepatic enzymes [102]. After cholesterol catabolism in the liver and their synthesis, BAs are metabolized by the gut microbiota. In the liver, they are conjugated with taurine or glycine amino acids, and the corresponding sodium or potassium salts are known as bile salts. Bile salts are usually stored in the gallbladder and secreted postprandially. Once in the gut, BAs and salts are metabolized further by the gut microbiome, to produce secondary BAs which undergo further re-absorption in a process known as enterohepatic recirculation. The types of BAs and salts exceed one hundred, including deoxycholic acid (DCA), ursodeoxycholic acid (UDCA) and lithocholic acid (LCA). They facilitate the gut uptake of many dietary lipids by their surfactant-like effects [103].

BAs are known to produce various biochemical and biological effects in the body, such as removal of cholesterol and bilirubin, emulsification and solubilization of lipid-soluble vitamins, facilitating their gut and oral uptake [104]. About 0.6 g of BAs/salts are metabolized and produced daily to replace lost BAs excreted in the feces. Among commonly researched BAs is the secondary BA, UDCA, which has been used for many centuries by traditional Chinese therapies [105]. UDCA is currently prescribed for cholestasis and liver cirrhosis [106]. UDCA's mechanism of action and pharmacology are anticipated to be because of its anti-inflammatory and cytoprotective effects [107].

### Encapsulation of stem cells

Encapsulation of stem cells (SC) is the latest advancement in encapsulation technology. SCs would be a resolution to our distressing deficiency of donated organs. However, encapsulating SCs is a fairly novel area of research, and this is explains why there are so few studies on encapsulated islets based on SCs for the adjustment of diabetes [108]. Researchers have used embryonic and adult SCs to provide islets for cell-replacement therapy. The ethical issues of using SCs for cell replacement have been widely discussed in the review article by Street *et al.* [109].

**Figure 2.**
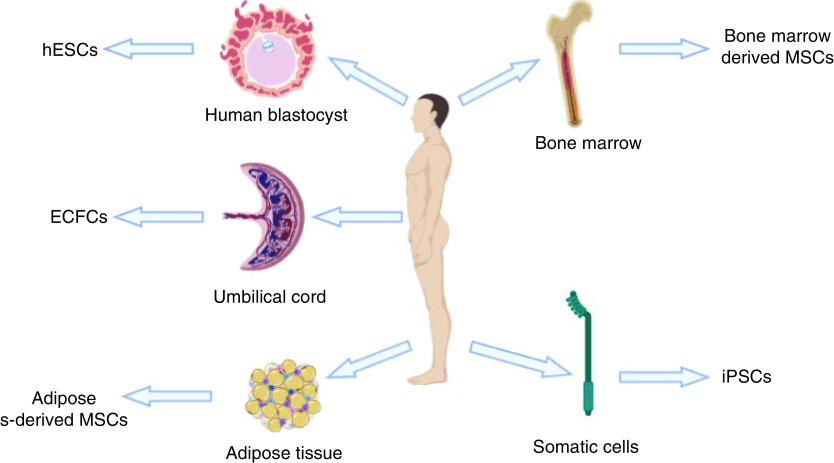
Sources of stem/progenitor cells. Umbilical cord blood can generate ECFCs; human blastocytes can generate hESCs; somatic cells can generate iPSCs; bone marrow can generate bone-marrow-derived mesenchymal stem cells; adipose tissue can generate adipose-derived mesenchymal stem cells. ECFC: Endothelial colony forming cell; hESC: Human embryonic stem cell; iPSC: Induced pluripotent stem cell; MSC: Mesenchymal stem cell.

Isolated islets in combination with mesenchymal SCs (MSCs), also known as adult SCs, can be isolated from different tissues such as bone marrow, somatic cells and adipose tissue (Figure 2). Isolated islets-co-MSCs have been explored by Shafiee *et al.* due to their potential to differentiate into different cell types. It was found that MSCs have the potential to produce paracrine factors promoting the function and growth of neighbouring cells. Pathak *et al.* have suggested bone marrow and adipocytes, as effective sources of MSCs, to be good options for allogenic MSC cotransplantation with isolated islets [110]. Micro-capsules of co-transplantations of islets with MSCs have been successful in mice, improving islet grafting [111]. This has encouraged many authors in the encapsulation of stem cells [112–114].

## Conclusion

Management of T1D using daily insulin injection might fail to achieve optimum glycemic control, and result in acute complications. Transplantations of whole pancreases or islets are presently considered potential options. Some authors have proposed protocols to define when to carry out islet or pancreas transplantation, and, in many scenarios, there is a degree of overlap. However, both methods have their pros and cons. Out of the two scenarios, ILT is considered the future and the more promising solution, therefore many strategies, including the encapsulation of islets andthe use of BAs or stem cells, are under investigation to overcome the limitations associated with ILT. Encapsulation of stem cells (SC) is the latest advancement in encapsulation technology in the past few years, and it seems to be encouraging, albeit still at the mouse level.

## Future perspective

ILT to treat diabetes have been widely researched for many years, and despite innovative ideas and dedicated researchers, no commercialable widely available product has made it to the market. The source of viable functional suitable islets remain challenging, and an appropriate matrices remain to be developed. Future perspective needs to focus on revolutionizing our approach to consider avenues of cell reprogramming, cell differentiation, bio-printing, gene editing, bio-nanotechnological scalable procedures that build on synthetic, semisynthetic and endogenously produced BAs and other compounds, and patient-specific approaches to enable the success of ILTs and wide applications in the clinic. Future studies need to focus on improved delivery systems and matrices that better resemble the human pancreas.

**Executive summary**

- Islet transplantation remains limited to Phase1/2 clinical trials with no widely used commercially available products.
- Main challenges to islet transplantation include body immune rejection and lack of appropriate delivery matrices.
- Bile acids have recently been proposed to complement matrices, suitable for islet transplantation.
- Bile acids are endogenously produced in humans and many are considered safe.
